# DNA-DAPI Interaction-Based Method for Cell Proliferation Rate Evaluation in 3D Structures

**DOI:** 10.3390/cimb43010021

**Published:** 2021-05-30

**Authors:** Egidijus Šimoliūnas, Paulius Kantakevičius, Miglė Kalvaitytė, Lina Bagdzevičiūtė, Milda Alksnė, Daiva Baltriukienė

**Affiliations:** 1 Life Sciences Center, Department of Biological Models, Institute of Biochemistry, Vilnius University, LT-10257 Vilnius, Lithuania; paulius.kantakevicius@gmail.com (P.K.); migle.kalvaityte@gmc.vu.lt (M.K.); lina.bagdzeviciute@cr.vu.lt (L.B.); milda.peciukaityte@gf.vu.lt (M.A.); daiva.baltriukiene@bchi.vu.lt (D.B.); 2 School of Biological Sciences, Faculty of Biology, Medicine and Health, The Univesity of Manchester, Manchester M13 9PL, UK

**Keywords:** cell number evaluation, 2D–3D environment, cell proliferation

## Abstract

Effective cell number monitoring throughout the three-dimensional (3D) scaffold is a key factor in tissue engineering. There are many methods developed to evaluate cell number in 2D environments; however, they often encounter limitations in 3D. Therefore, there is a demand for reliable methods to measure cell proliferation in 3D surroundings. Here, we report a novel technique for the DNA content-based evaluation of cell proliferation using DNA-binding dye DAPI. We demonstrated the method’s compatibility with four different cell cultures: cancer lines MCF-7 and MH-22a, embryonic fibroblast cell line Swiss 3T3, and primary mesenchymal stem cell culture isolated from rat’s incisors. The DAPI based method was able to successfully evaluate cell proliferation in 2D, 2.5D, and 3D environments. Even though the proposed method does not discriminate between viable and dead cells, it might give a convenient snapshot of the cell number at a given time point. This should help to more reliably evaluate various processes proceeding in 2.5D and 3D cultures.

## 1. Introduction

Cell proliferation is a process that results in an increase in cell number due to cell division and growth. The rate of cell proliferation provides valuable information about their basic health and maintenance, as well as their responses to a particular drug or a disease state. Therefore, it is a good indicator of the overall cell wellbeing [1,2]. A number of assays have been developed for analyzing cell growth by performing cell counts or by monitoring DNA content, DNA synthesis, metabolic activity, or protease activity [2,3,4,5,6]. These methods have varying levels of sensitivity, reproducibility, and compatibility with high-throughput formatting [7]. The choice of the relevant method depends on the type of cells/tissues.

Tissue engineering encompasses the study of cell interaction with three-dimensional (3D) scaffolds. These 3D scaffolds are generally highly porous structures to ensure cell integration, nutrient and oxygen diffusion, and waste removal [8,9]. Effective cell seeding and cell integration throughout the 3D environment play an important role in tissue engineering. The rate of cell proliferation inside the 3D scaffolds shows their integration and biocompatibility [10]. To accurately track cell-scaffold interaction processes, sometimes it is necessary to know the exact cell number [11,12]. However, current available methods for measuring cell proliferation are not accurate enough to evaluate the cell number in 3D surroundings [13,14]. Divieto and Sassi proposed a novel approach for the quantitative assessment of cell viability in 3D surroundings based on enzymatic reduction of resazurin dye by viable cells [15]. However, this method is not applicable in certain cases as there is a broad range of synthetic materials, which can be used for scaffold fabrication. Due to chemical interactions with resazurin reagents, some of the materials might produce artefacts, which could interfere with results acquisition, while other materials are known to be auto-fluorescent [16,17]. Therefore, they might reduce the sensitivity of the resazurin assay. Additionally, it should be taken into consideration that contact inhibition of proliferation or onset of differentiation can change the metabolic activity of cells, which can lead to differences in resazurin metabolism at different time points [18]. This also applies to other popular cell number evaluation methods, which are based on cell metabolic activity, for example, MTT [19,20]. Thus, in cases when cells cannot be counted directly, or cellular metabolism might directly influence cell number measurements, other types of methods have to be used [21,22,23,24].

To perform a precise evaluation of cell number in a scaffold, several different strategies can be applied. One of the them is based on the measurement of cellular protein content, e.g., using sulforhodamine B, the quantification of intracellular proteins release (glucose-6-phosphate dehydrogenase and lactate dehydrogenase) [25,26,27]. The mentioned methods are not as sensitive to external or internal factors as MTT and resazurin-based methods. Nevertheless, in cell differentiation studies, the cell proteome can change significantly; thus, their use is limited [20,27]. The other strategy is based on the quantification of cellular DNA and can measure cell number at the population level or even at the individual cell level [28]. These methods are generally based on stoichiometric fluorophore binding to DNA, meaning that cell number can be evaluated using fluorescent microscopy, PCR quantification, or fluorescence measurements [28,29,30]. Only several fluorophores are usually used for cell number evaluation in 2D and 3D—PicoGreen, CyQuant, and Hoechst 33258. As known to the authors, DAPI (4′,6-diamidino-2-phenylindole), which is extensively used for staining cell nucleus, has not yet been applied for cell number evaluation in 3D environments [28,31,32,33]. Thus, here, we report a DAPI-based DNA content measuring assay for cell quantification in 2D and 3D surroundings.

## 2. Methods

### 2.1. Materials

PBS (09-8912-100, Medicago, Uppsala, Sweden), SDS (101161470, Sigma-Aldrich, Taufkirchen, Germany), Triton X-100 (X100-5ML, Sigma-Aldrich), SSC (93017-10L-F, Sigma-Aldrich), DAPI (D1306, Invitrogen, Carlsbad, CA, USA), IMDM (42200-030, Gibco, Dublin, Ireland), DMEM (12800-017, Gibco), Penicillin/Streptavidin (15140-122, Gibco), Fetal bovine serum (A31608-01, Gibco), Isopropanol (UN1219, VWR international GmbH, Darmstadt, Germany), Ethanol (32221-2.5L, Sigma-Aldrich), Sodium citrate (18996-35-5, Sigma-Aldrich), Paraformaldehyde (30525-89-4, Sigma-Aldrich), BSA (9048-46-8, Sigma-Aldrich), TRITC conjugated Rhodamine Phalloidin (90228, Millipore), HEPES (15630-056, Gibco), TRIzol (15596026, Invitrogen), and Polylactic acid (PLA) (Pure PLA filament, Ultimaker, Utrecht, The Netherlands).

### 2.2. Cell Culture

Three commercially available cell lines—MCF-7, MH-22a, Swiss 3T3 and primary dental pulp-derived stem cells (DPSCs) isolated from Wistar rat—were used for experiments. MH-22a cells were obtained from the Institute of Cytology, Sankt-Petersburg, Russian Federation. MCF-7 cells were obtained from the Cell line Service GmbH (Eppelheim, Germany). Swiss 3T3 fibroblasts were obtained from ATCC (Kielpin, Poland) and were a kind gift from dr. Mindaugas Valius. DPSC isolation was performed as described in our previous protocol^12^. Cancer cell lines, MCF-7 and MH-22a, were grown in DMEM growth medium supplemented with 10% fetal bovine serum and 1% streptavidin/penicillin. Swiss 3T3 and DPSC were grown in IMDM medium, supplemented with 10% fetal bovine serum and 1% streptavidin/penicillin. Cell number for the experiments was evaluated by Scepter Cell Counter (Milipore, Burlington, MA, USA) according to the manufacturer’s instructions.

### 2.3. Scaffolds Printing

Two types of scaffolds were used in this research—wavy and porous. Scaffolds were produced from pure PLA filament (Ultimaker B.V.) and printed with FFF 3D printer “Ultimaker” Original (Ultimaker B.V.) as described previously [34]. The printer nozzle had a diameter of 400 µm, the printing was done at 180–190 °C, and the pinpoint scanning velocity was set to 30 mm/s. CAD models of wavy and porous scaffolds were produced with Fusion 360 (Autodesk Inc., San Rafael, CA, USA). Each layer of the 3D porous structure was composed of threads whose width and height were 500 μm and 400 μm. The structure had four layers each rotated by 90 degrees, and the height of the final scaffold was 1600 µm. Wavy scaffolds were printed in a standing up position. Layer height was 188 µm and formed hills were around 250 µm in height. The total height of the printed wavy scaffold was around 1000 µm.

### 2.4. Assessment of the Background Fluorescence of Lysis Buffers

Two types of lysis buffers containing either SDS or Triton X-100 detergent were tested. Lysis buffer composition was the following: saline-sodium citrate (SSC) buffer (pH 7.2) supplemented with SDS or Triton X-100 to the final concentration of 0.01, 0.015, 0.02, 0.025, 0.03, 0.035, and 0.04% (*w/v*). DAPI fluorescent dye was diluted in the SSC buffer to the concentration of 4 µg/mL. In addition, 100 µL of lysis buffer was mixed with 100 µL DAPI dye solution and incubated at 37 °C in the dark for 30 min. Next, DAPI fluorescence was measured with the Varioskan Flash Multimode Reader (Thermo Scientific, Waltham, MA, USA). Fluorometric measurements were made with excitation at 360 nm and emission at 460 nm.

### 2.5. Fluorescence Emission Sweep

DAPI background fluorescence and fluorescence in the presence of DNA was assessed by a fluorescence emission sweep with the Thermo Scientific Varioskan Flash Multimode Reader. In addition, 100 µL 0.02% SDS in SSC buffer was mixed with 100 µL of 0.2, 0.8, 1.6, 3.2, and 4 µg/mL DAPI solutions in SSC buffer. In the presence of DNA, 5, 10, 20, 75, and 150 ng/mL concentrations of DNA were used. DNA was isolated from the MCF-7 cell line using TRIzol reagent according to the manufacturer’s instructions. After incubation at 37 °C in the dark for 30 min, fluorescence emission sweep was performed, starting from 400 nm and ending at 600 nm wavelength.

### 2.6. Immunofluorescence Staining

The ability of lysis buffers to dissolve cells was evaluated by immunofluorescence staining of cell cytoskeleton and nuclei. DPSC were seeded in 48-well plates at a concentration of 40,000 cells/mL. The next day, cells were treated with lysis buffers containing 0, 0.02, or 0.04% of either SDS or Triton X-100 and incubated for 30 min at 37 °C temperature. Then, buffers were removed and cells were washed twice with PBS. Next, cells were fixed with 4% paraformaldehyde for 15 min at RT and washed once with PBS, followed by incubation with 1 mg/mL BSA in PBS for 1 h at RT. Then, they were washed again once with PBS and 200 μL of 0.6 µg/mL TRITC conjugated Rhodamine Phalloidin and 0.2 µg/mL DAPI solution in PBS was added and incubated at RT in the dark for 30 min. After incubation, wells were washed three times with PBS for 30 s each wash. Cells must be covered with PBS prior to visualization with a fluorescent microscope to prevent cells from drying out.

### 2.7. Calibration Curve

For MCF-7, MH-22a and Swiss 3T3 calibration curve experiments cells were seeded in 24-well plates at densities: 250, 500, 750, 1000, 2500, 5000, 7500, 10,000, 25,000, 50,000, and 100,000 cells/well. For the DPSC calibration curve, densities of 100, 250, 500, 750, 1000, 2500, 5000, 7500, 10,000, 25,000, 50,000 and 100,000 cells/well were used. After MCF-7, MH-22a, Swiss 3T3, and DPSC cells attached to the plate surface, they were washed twice with PBS and incubated for 1 h or overnight at –20 °C temperature. After incubation at −20 °C, cells were treated with 0.02% SSD in SSC buffer and incubated at 37 °C while shaking at 300 RPM for 30 min. The required amount of lysis buffer depends on the cell growth surface area and should be determined for every cell line. In this case, all the cells were lysed with 400 µL of lysis buffer. Then, 100 µL of lysate was mixed with 100 µL of 4 µg/mL DAPI fluorescent dye diluted in SSC to a final DAPI concentration of 2 µg/mL, and incubated at 37 °C in the dark for 30 min. Finally, DAPI fluorescence was measured with the Varioskan Flash Multimode Reader. Fluorometric measurements were made with excitation at 360 nm and emission at 460 nm. The background fluorescence values were subtracted from the data.

If cells are not completely dissolved by the 0.02% SDS lysis buffer, a larger concentration of lysis buffer should be used. In such case, additional dilutions would be needed since the final SDS concentration has to be 0.01%. To measure smaller cell numbers, a lower DAPI concentration should be applied.

### 2.8. Assessment of the Cell Number in 3D Surroundings

DPSC cells were seeded onto three different surfaces: 2D 24-well plate plastic surface, 2.5D wavy PLA surface, and 3D porous PLA surface. Cell numbers for seeding were calculated according to the surface area—15,000 cells/cm^2^. In addition, 30,000 DPSC cells were seeded into each well of the 24-well plate and 15,000 cells to wavy and porous PLA surfaces as their surface areas were assumed to be 1 cm^2^. Cell numbers were evaluated after 3, 24, 48, 72, 96, and 120 h. To measure cell numbers, the method described above in Section 2.7 was used.

### 2.9. Statistical Analysis

Linear regression models were used to calculate the slopes of the calibration curves. For data analysis and visualization, the R statistics program was used (version 3.5.1) with ggplot2 library functions. Data in graphs are shown as dots with ± SD while fluorometric scans are shown as line graphs. Linear regression models are shown as black lines with a grey area indicating a 95% confidence interval of the model. The goodness of fit was evaluated by calculating a coefficient of determination. Each data point was acquired from at least three independent samples.

## 3. Results

### 3.1. Assessment of Lysis Buffer Composition

**Figure 1:**
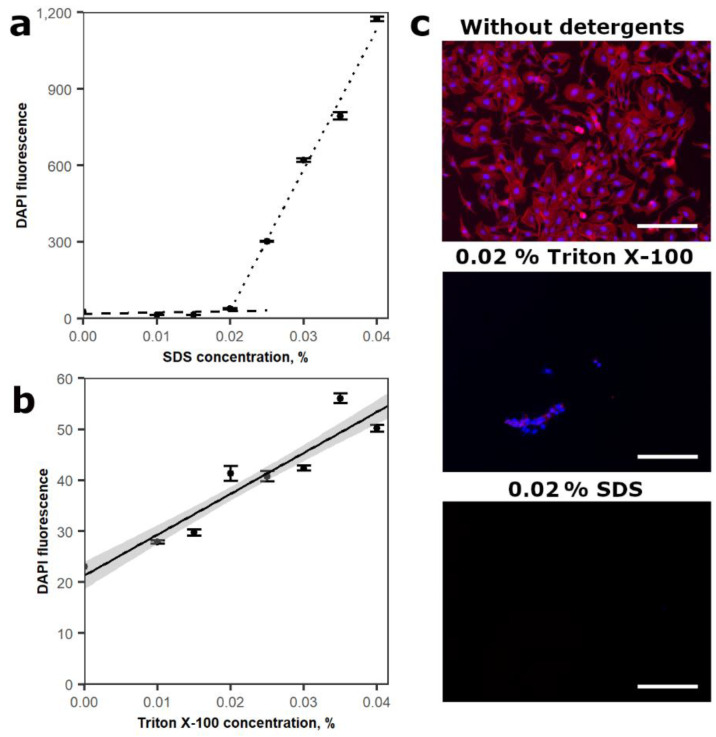
Assessment of efficiency of cell lysis buffers. (**a**) SDS concentration-dependent 2 µg/mL DAPI fluorescence; (**b**) Triton X-100 concentration-dependent 2 µg/mL DAPI fluorescence; (**c**) evaluation of the ability of SDS or Triton X-100 containing lysis buffers to dissolve DPSC cells. Immunofluorescence pictures depict cell nuclei (blue) and actin filaments (red). Values in graphs are shown as mean ± SD. *n* = 3, scale bar marks 200 µm.

Two types of cell lysis buffers based on either SDS or Triton X-100 detergents were evaluated for effective cell lysis and nuclear envelope dissolution. SDS is an anionic detergent while Triton X-100 is nonionic. First, the interaction between lysis buffers with different detergent concentrations and DAPI were assessed (Figure 1a,b). For this purpose, SSC buffer (pH 7.2), containing 0.01, 0.015, 0.02, 0.025, 0.03, 0.035, 0.04% SDS or Triton X-100 and 2 µg/mL of DAPI dye, was used. After evaluation of DAPI fluorescence, a linear relationship between DAPI fluorescence and Triton X-100 concentrations ranging from 0.01 to 0.04% was observed. However, in buffers with SDS anionic detergent, two different linear processes were determined. From 0.01 to 0.02%, DAPI fluorescence increased gradually; however, if SDS concentration was higher than 0.02%, a rapid increase in DAPI fluorescence was observed. Thus, a low concentration of SDS should be used in the lysis buffer. In the case of Triton X-100, a wide range of detergent concentrations can be used for cell lysis as it would not interfere with DAPI fluorescence measurements.

Most DNA in the cell are localized in the nucleus; thus, the ability of lysis buffers to effectively dissolve cellular and nuclear membranes were evaluated by immunocytochemical analysis (Figure 1c). DPSC was chosen for this experiment since it has a dense cytoskeletal network which could interfere with the nucleus lysis (Figure 1c). In addition, 40,000 cells/mL were seeded into 48-well plates and treated with 0.02 and 0.04% SDS or Triton X-100-containing lysis buffers. After 1 h incubation with lysis buffers, the cells were washed and the cytoskeleton and cell nuclei were stained with Rhodamine Phalloidin and DAPI, respectively. Both SDS concentrations effectively dissolved the cells as no cells or nuclei were left. On the contrary, 0.02% Triton X-100 was unable to dissolve the cells completely as some cells and nuclei were still visible after 1 h incubation. However, 0.04% Triton X-100 containing a lysis buffer completely dissolved cells and nuclei. Consequently, lower concentrations of Triton X-100 detergent are unsuitable for dissolving cell nuclei. Furthermore, Triton X-100 produced a stronger background signal compared to the SDS lysis buffer, which can influence the sensitivity of this method. Based on these data, anionic detergent SDS was chosen for cell lysis in further experiments.

### 3.2. Determination of DAPI Working Concentration

**Figure 2:**
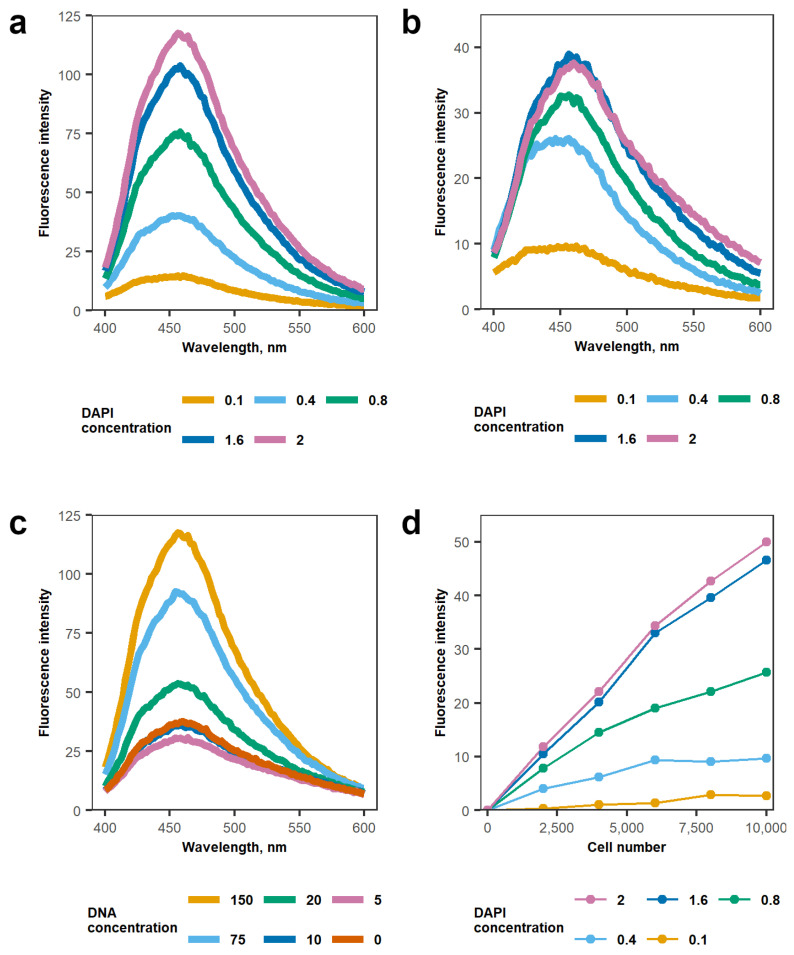
Evaluation of optimal working concentrations of DAPI. Fluorescence signals of: (**a**) various DAPI concentrations: 0.1, 0.4, 0.8, 1.6, and 2 µg/mL with 150 ng/mL DNA; (**b**) the same concentrations of DAPI, but without DNA; (**c**) 2 µg/mL DAPI with various DNA concentrations: 5, 10, 20, 75, and 150 ng/mL; (**d**) exposure of MH-22a cells to the different DAPI concentrations: 0.1, 0.4, 0.8, 1.6, and 2 µg/mL. (**a**–**c**) line graphs depict fluorescence sweep data, (**d**) curves represent DNA-DAPI fluorescence intensity values. Data for a, b, c, and d were obtained from three independent samples.

Next, DAPI concentration-dependent fluorescence in SDS-containing lysis buffer was assessed. The final SDS concentration in the sample after cell lysis and addition of DAPI was 0.01%, as it was shown to have the least effect on the background DAPI fluorescence (Figure 1a). A wide range of DAPI concentrations was evaluated in 0.01% SDS containing lysis buffer—0.1, 0.4, 0.8, 1.6, and 2 µg/mL (Figure 2a,b). DAPI fluorescence emission sweep was performed ranging from 400 to 600 nm. Logarithmic fluorescence intensity dependence on DAPI concentration was observed (Figure 2b). The same tendency was observed with 150 ng/mL of DNA present in the solution (Figure 2a). The addition of DNA increased the fluorescence intensity compared to DAPI only solution; however, the intensity was still dependent on DAPI concentration—larger DAPI concentrations produced a stronger signal. Nevertheless, DNA-DAPI complex fluorescence intensity at small DNA concentrations was changing nonlinearly (Figure 2c). This was evaluated by measuring 2 µg/mL DAPI fluorescence emission sweep from 400 to 600 nm with different DNA concentrations—5, 10, 20, 75, and 150 ng/mL. DAPI fluorescence intensity without DNA was greater than in combination with 5 ng/mL DNA. However, around 10 ng/mL DNA concentration, the intensity equalized to DAPI background fluorescence intensity and increased logarithmically from there.

Next, the effect of dye concentration ranging from 0.1 to 2 µg/mL on the dynamic range of MH-22a cell number evaluation was determined (Figure 2d). The use of 2 µg/mL DAPI allowed for accurately quantifying large amounts of DNA in the sample. However, with increasing dye concentrations, the sensitivity to smaller cell concentrations reduces due to an increase in the background signal; thus, for smaller cell concentrations, lower amounts of DAPI should be used. Likewise, at small DNA concentrations, the nonlinear processes might inhibit accurate cell number measurements. These processes were not observed with higher MH-22a cell numbers.

### 3.3. Assessment of the Method with Various Cell Lines

**Figure 3:**
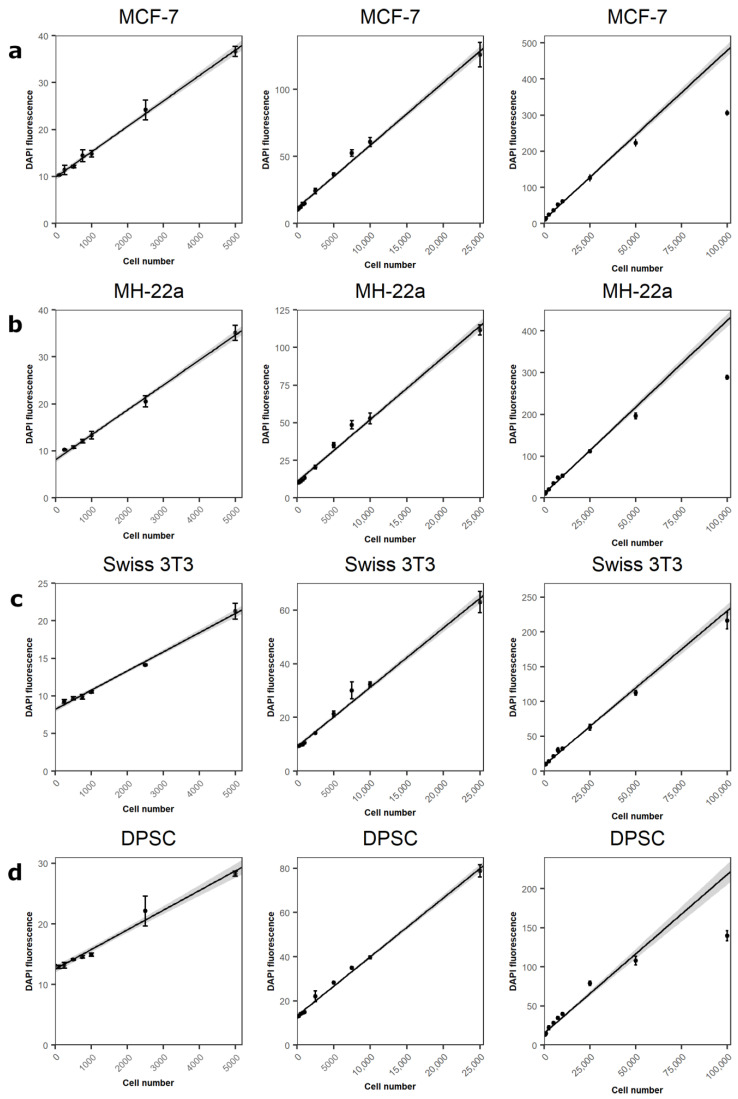
Dependence of DAPI fluorescence on the cell number. Cell numbers range from 200 to 5000 in the first column, from 200 to 25,000 in the second, and from 200 to 100,000 in the third. (**a**) MCF-7; (**b**) MH-22a; (**c**) Swiss 3T3 cell lines; (**d**) dental pulp primary stem cells (DPSC). Values in graphs are shown as mean ± SD. Lines are linear regression models comprised from the linear part of the graph—from 200 to 25,000 cells lysate DAPI fluorescence intensity data. *n* = 3.

Finally, the optimal lysis buffer composition and concentration of DAPI were tested on four cell lines—cancer lines MCF-7 and MH-22a, mouse embryo fibroblast stem cell line Swiss 3T3, and primary rat DPSC (Figure 3). All tested cell types revealed a linear relationship between DAPI fluorescence intensity and cell numbers. MCF-7 and MH-22a cancer lines displayed the largest DAPI fluorescence intensity at 100,000 cell count, where DAPI fluorescence was at 306 and 288 intensity units, respectively (Figure 3a,b). However, a linear relationship between DAPI fluorescence intensity and cell number was observed from 250 to 25,000 cells (Table 1). In this range, the determined coefficient of correlation (R^2^) was strong (0.988) for both cell lines. Different sensitivity was observed with the Swiss 3T3 cell line (Figure 3c). A strong linear relationship was shown from 250 to 100,000 cells. However, compared to cancer cell lines, at the highest Swiss 3T3 count—100,000, DAPI fluorescence intensity was considerably lower (216 arbitrary intensity units). The coefficient of correlation in the range from 250 to 25,000 cells was also strong—0.986 (Table 1). In the case of primary stem cells, the linear relationship was detected only in the range of 100 to 25,000 cells (Figure 3d). Observed signal strength was substantially lower at 100,000 cells compared to both cancer and Swiss 3T3 cell lines (139 arbitrary intensity units). However, the determined R^2^ was very strong—0.996 (Table 1).

Linear regression analysis showed different slope coefficients (k) for different cell lines. Two cancer lines had similar coefficient values—0.00468 and 0.00414 for MCF-7 and MH22a cell lines, respectively (Table 1). Swiss 3T3 and DPSC k values were lower than cancer cell lines—0.0021 and 0.0026, respectively.

### 3.4. Assessment of the Method in 2D, 2.5D, and 3D Environments

**Figure 4:**
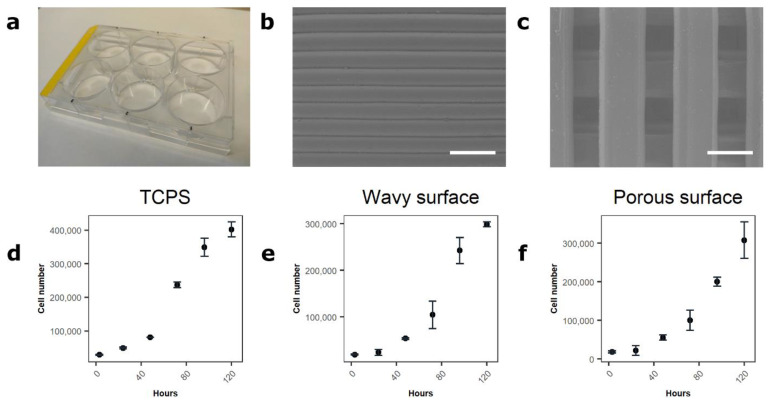
Assessment of cell proliferation on different surfaces. (**a**–**c**) tissue culture plate surface (TCPS), wavy and porous scaffolds, respectively; (**d**) DPSC proliferation on TCPS; (**e**) DPSC proliferation on 2.5D wavy surface; (**f**) DPSC proliferation on 3D porous scaffolds. Scale bars in (**b**,**c**) represent 500 µm. Values in graphs are shown as mean ± SD. Five samples were used for each time point.

The proposed technique was applied for evaluating the proliferation of DPSC grown on three different surfaces—tissue culture plate surface (TCPS) (Figure 4a), PLA scaffold with wavy topography (Figure 4b), and PLA porous 3D scaffold (Figure 4c). In addition, 15,000 cells/cm^2^ were seeded onto these surfaces and the change in cell number was observed after 3, 24, 48, 72, 96, and 120 h (Figure 4d–f). After 3 h, the anticipated numbers of DPSC were detected on the tested surfaces, 28,756 ± 1504 cells on plastic 24-well TCPS, 19,373 ± 1093 cells on the wavy PLA surface, and 18413 ± 2661 cells on the porous PLA surface. After attachment to the surfaces, the cells started to proliferate and, after 120 h, the cells reached 401,906 ± 22,135 on the plastic surface, 298,411 ± 5076 on the PLA wavy surface, and 307,498 ± 47,271 on the PLA porous surface.

## 4. Discussion

Evaluation of cell number in the 3D microenvironment is an important factor ensuring the success of tissue engineering as it is critical to know how many cells generate a specific signal to correctly evaluate the cell response [1]. There are many methods developed for measuring cell number in 2D, which are based on cell metabolism [3], cell-depended biomolecule concentration [4,5], or cell count [5]. However, the vast majority of these conventional methods are not compatible with 3D surroundings [14,21]. Gantenbein-Ritter et al. compared the accuracy of trypan blue staining, lactate dehydrogenase assay, and calcein AM/ethidium homodimer-1 staining [13]. They concluded that these assays are applicable for accurate cell viability/proliferation evaluation in the 3D environment. However, it should be noted that the authors performed the evaluation of cell viability after performing fibrin digestion. Methods that involve a digestion step tend to selectively digest dead cells, which lead to overestimation of cell viability in any downstream protocol. Another group optimized a resazurin assay for 3D cultures [15]. In the latter case, a non-biodegradable highly porous Bioral scaffold was used in the analysis. However, the resazurin-based assays are dependent on the cell metabolic state, and it is known that, in various conditions, cell metabolism can change, thus over- or under-estimation of the overall cell population might occur [18]. Assessment of the entire cellular DNA content is becoming a reliable method for cell proliferation analysis [28,29,30]. The development of a 3D compatible DNA concentration-based method was the goal of this study. It is known that cells can vary in size and their nuclear DNA content [35]. Therefore, we have analyzed four different types of cells in this study to evaluate a wide range of different lengths of genomes (human, rat, and mouse) and cancer cells which are known to have polyploid genomes [36]. MCF-7 and MH-22a are of cancerous origin; meanwhile, Swiss 3T3 and DPSCs are derived from healthy mouse and rat tissues, respectively.

Two types of cell lysis buffers were evaluated: SDS and Triton X-100. Both lysis buffers had an impact on DAPI fluorescence in the absence of DNA. However, SDS lysis buffer with concentrations greater than 0.02% had dramatically higher DAPI fluorescence. SDS is known to form micelles at certain SDS concentration depending on the solution properties [37]. Inside these micelles, hydrophobic conditions, which could increase DAPI fluorescence intensity, are formed [38]. Triton X-100 concentration in a lysis buffer had a linear relationship with DAPI fluorescence intensity, thus a critical micelle forming concentration was not reached in this case.

DAPI is a DNA specific dye. Upon binding to DNA, its excitation coefficient increases ~20 times, however, when bound to RNA, it remains much smaller and the emission maximum shifts to about 500 nm [16,17]. This DAPI property makes it a great dye for DNA staining. However, at small DNA concentrations, it is difficult to quantify DNA in samples, since a nonlinear relationship reduces the detectable DNA concentration range. Nevertheless, the Swiss 3T3 cell line presented a strong linear relationship from 200 cells to almost 100,000 cells. Such an interval of detectable cells is wide enough and could be adopted to many experimental designs in which cell cultures are used. However, it was noticed that the same number of different types of cells produces different fluorescence intensity results. In the case of human and mouse cancer cells, the difference was inconsiderable; however, the difference between cancer cells and stem cells was apparent. This might have been caused by differences in genome length and DNA copy numbers [39,40]. However, the optimal range of detectable cells is cell type dependent and should be determined individually prior to use.

The proposed method does not discriminate between viable and dead cells. Nevertheless, it can be used for cell number determination inside 3D scaffolds. This method was used to evaluate DPSC proliferation on various surfaces (2D TCPS, 2.5D wavy PLA, and 3D porous PLA scaffolds). The selected lysis buffer was capable of dissolving cells and their DNA, thus allowing us to successfully determine cell numbers in 2D, 2.5D, and even 3D environments. Moreover, the proposed method is independent of cellular metabolic states and could be applied for the quantification of less metabolically active cells.

Additionally, our results showed that, by combining two different lysis buffers, cells can be lysed in two phases; the first cell membrane with 0.02% Triton X-100, and later complete cell lysis with 0.02% SDS containing lysis buffer. This is important when combining two different types of assays with one sample—for example, measuring alkaline phosphatase (ALP) activity where cell lysis is required to extract cytosolic ALP [41]. ALP activity assay is often combined with overall protein content measurements for ALP activity standardization in different samples. However, it could be done with our DAPI based method as well.

## 5. Conclusions

We developed a method for cell quantification by measuring DNA content. This method can be applied to the cells grown in 2D, 2.5D, and 3D environments. It is applicable for the quantification of healthy, cancerous, and stem cell numbers. Since this DAPI based method analyzes cellular DNA content, its accuracy is not affected by the changes in cell metabolism due to differentiation or other biological processes. Its quantification range depends on the cell type. However, we were able to perform accurate measurements from 250 to 100,000 cells with MCF-7, MH-22a, Swiss 3T3, and DPSC. This method uses simple buffers and a common DNA binding dye DAPI, which can be combined with different assays where signal standardization to the cell number is required.

## Figures and Tables

**Table 1:** Linear regression equation constants and model accuracy. Values have been determined by analyzing DAPI fluorescence intensity dependence to cell number data ranging from 250 to 25,000 cells. Equation: y = kx + b.

Cell line	k	b	R^2^
MCF-7	0.0047	11.6	0.988
MH-22a	0.0041	10.9	0.988
Swiss 3T3	0.0021	9.1	0.986
DPSC	0.0026	13.6	0.996

## Data Availability

Not applicable.

## References

[1] Knapp B., Rebhan I., Kumar A., Matula P., Kiani N.A., Binder M., Erfle H., Rohr K., Eils R., Bartenschlager R. (2011). Normalizing for individual cell population context in the analysis of high-content cellular screens. BMC Bioinform..

[2] Kim S., Choung S., Sun R.X., Ung N., Hashemi N., Fong E.J., Lau R., Spiller E., Gasho J., Foo J. (2020). Comparison of Cell and Organoid-Level Analysis of Patient-Derived 3D Organoids to Evaluate Tumor Cell Growth Dynamics and Drug Response. SLAS Discov..

[3] Śliwka L., Wiktorska K., Suchocki P., Milczarek M., Mielczarek S., Lubelska K., Cierpiał T., Łyżwa P., Kiełbasiński P., Jaromin A. (2016). The Comparison of MTT and CVS Assays for the Assessment of Anticancer Agent Interactions. PLoS ONE.

[4] Ghali O., Broux O., Falgayrac G., Haren N., Van Leeuwen J., Penel G., Hardouin P., Chauveau C. (2015). Dexamethasone in osteogenic medium strongly induces adipocyte differentiation of mouse bone marrow stromal cells and increases osteoblast differentiation. BMC Cell Biol..

[5] Lee Y., Kim B.-Y., Choi S. (2018). On-Chip Cell Staining and Counting Platform for the Rapid Detection of Blood Cells in Cerebrospinal Fluid. Sensors.

[6] Li W., Zhou J., Xu Y. (2015). Study of the in vitro cytotoxicity testing of medical devices (Review). Biomed. Rep..

[7] Romar G.A., Kupper T.S., DiVito S.J. (2016). Research Techniques Made Simple: Techniques to Assess Cell Proliferation. J. Investig. Dermatol..

[8] Loh Q.L., Choong C. (2013). Three-Dimensional Scaffolds for Tissue Engineering Applications: Role of Porosity and Pore Size. Tissue Eng. Part B Rev..

[9] Neto A.S., Ferreira J.M.F. (2018). Synthetic and Marine-Derived Porous Scaffolds for Bone Tissue Engineering. Materials.

[10] Abbasi N., Hamlet S., Love R.M., Nguyen N.-T. (2020). Porous scaffolds for bone regeneration. J. Sci. Adv. Mater. Devices.

[11] Chen Z., Yan X., Yin S., Liu L., Liu X., Zhao G., Ma W., Qi W., Ren Z., Liao H. (2020). Influence of the pore size and porosity of selective laser melted Ti6Al4V ELI porous scaffold on cell proliferation, osteogenesis and bone ingrowth. Mater. Sci. Eng. C.

[12] Alksne M., Simoliunas E., Kalvaityte M., Skliutas E., Rinkunaite I., Gendviliene I., Baltriukiene D., Rutkunas V., Bukelskiene V. (2019). The effect of larger than cell diameter polylactic acid surface patterns on osteogenic differentiation of rat dental pulp stem cells. J. Biomed. Mater. Res. Part A.

[13] Gantenbein-Ritter B., Potier E., Zeiter S., Van Der Werf M., Sprecher C.M., Ito K. (2008). Accuracy of Three Techniques to Determine Cell Viability in 3D Tissues or Scaffolds. Tissue Eng. Part C Methods.

[14] Pan Y., Hu N., Wei X., Gong L., Zhang B., Wan H., Wang P. (2019). 3D cell-based biosensor for cell viability and drug assessment by 3D electric cell/matrigel-substrate impedance sensing. Biosens. Bioelectron..

[15] Divieto C., Sassi M.P. (2015). A first approach to evaluate the cell dose in highly porous scaffolds by using a nondestructive metabolic method. Futur. Sci. OA.

[16] Riss T.L., Moravec R.A., Niles A.L., Duellman S., Benink H.A., Worzella T.J., Minor L. (2016). Cell Viability Assays. Assay Guidance Manual.

[17] Qi L., Knapton E.K., Zhang X., Zhang T., Gu C., Zhao Y. (2017). Pre-culture Sudan Black B treatment suppresses autofluorescence signals emitted from polymer tissue scaffolds. Sci. Rep..

[18] Li Q., Gao Z., Chen Y., Guan M.-X. (2017). The role of mitochondria in osteogenic, adipogenic and chondrogenic differentiation of mesenchymal stem cells. Protein Cell.

[19] Kumar P., Nagarajan A., Uchil P.D. (2018). Analysis of Cell Viability by the MTT Assay. Cold Spring Harb. Protoc..

[20] Stepanenko A.A., Dmitrenko V.V. (2015). Pitfalls of the MTT assay: Direct and off-target effects of inhibitors can result in over/underestimation of cell viability. Gene.

[21] Forsey R.W., Chaudhuri J.B. (2009). Validity of DNA analysis to determine cell numbers in tissue engineering scaffolds. Biotechnol. Lett..

[22] Silva L.P., Lorenzi P.L., Purwaha P., Yong V., Hawke D.H., Weinstein J.N. (2013). Measurement of DNA Concentration as a Normalization Strategy for Metabolomic Data from Adherent Cell Lines. Anal. Chem..

[23] Hayton S., Maker G.L., Mullaney I., Trengove R.D. (2017). Experimental design and reporting standards for metabolomics studies of mammalian cell lines. Cell. Mol. Life Sci..

[24] Ng K.W., Leong D.T., Hutmacher D.W. (2005). The Challenge to Measure Cell Proliferation in Two and Three Dimensions. Tissue Eng..

[25] Orellana E.A., Kasinski A.L. (2016). Sulforhodamine B (SRB) Assay in Cell Culture to Investigate Cell Proliferation. Bio-Protocol.

[26] Zhu A., Romero R., Petty H.R. (2011). An enzymatic colorimetric assay for glucose-6-phosphate. Anal. Biochem..

[27] Kaja S., Payne A.J., Naumchuk Y., Koulen P. (2017). Quantification of Lactate Dehydrogenase for Cell Viability Testing Using Cell Lines and Primary Cultured Astrocytes. Curr. Protoc. Toxicol..

[28] Gomes C.J., Harman M.W., Centuori S.M., Wolgemuth C.W., Martinez J.D. (2018). Measuring DNA content in live cells by fluorescence microscopy. Cell Div..

[29] Ruoß M., Kieber V., Rebholz S., Linnemann C., Rinderknecht H., Häussling V., Häcker M., Damink L.H.H.O., Ehnert S., Nussler A.K. (2019). Cell-Type-Specific Quantification of a Scaffold-Based 3D Liver Co-Culture. Methods Protoc..

[30] Rivero R.E., Capella V., Liaudat A.C., Bosch P., Barbero C.A., Rodríguez N., Rivarola C.R. (2020). Mechanical and physicochemical behavior of a 3D hydrogel scaffold during cell growth and proliferation. RSC Adv..

[31] Quent V.M., Loessner D.L., Friis T.E., Reichert J.C., Hutmacher D.W. (2010). Discrepancies between metabolic activity and DNA content as tool to assess cell proliferation in cancer research. J. Cell. Mol. Med..

[32] Nava M.M., Miroshnikova Y.A., Biggs L.C., Whitefield D.B., Metge F., Boucas J., Vihinen H., Jokitalo E., Li X., Arcos J.M.G. (2020). Heterochromatin-Driven Nuclear Softening Protects the Genome against Mechanical Stress-Induced Damage. Cell.

[33] Ligasová A., Koberna K. (2019). Quantification of fixed adherent cells using a strong enhancer of the fluorescence of DNA dyes. Sci. Rep..

[34] Malinauskas M., Rekštytė S., Lukoševičius L., Butkus S., Balčiūnas E., Pečiukaitytė M., Baltriukienė D., Bukelskienė V., Butkevičius A., Kucevičius P. (2014). 3D Microporous Scaffolds Manufactured via Combination of Fused Filament Fabrication and Direct Laser Writing Ablation. Micromachines.

[35] Gillooly J.F., Hein A., Damiani R. (2015). Nuclear DNA Content Varies with Cell Size across Human Cell Types. Cold Spring Harb. Perspect. Biol..

[36] Mirzayans R., Andrais B., Murray D. (2018). Roles of Polyploid/Multinucleated Giant Cancer Cells in Metastasis and Disease Relapse Following Anticancer Treatment. Cancers.

[37] Bhaisare M.L., Pandey S., Khan M.S., Talib A., Wu H.-F. (2015). Fluorophotometric determination of critical micelle concentration (CMC) of ionic and non-ionic surfactants with carbon dots via Stokes shift. Talanta.

[38] Favilla R., Parisoli A., Mazzini A. (1997). Alkaline denaturation and partial refolding of pepsin investigated with DAPI as an extrinsic probe. Biophys. Chem..

[39] Shlien A., Malkin D. (2009). Copy number variations and cancer. Genome Med..

[40] Emes R.D., Goodstadt L., Winter E.E., Ponting C.P. (2003). Comparison of the genomes of human and mouse lays the foundation of genome zoology. Hum. Mol. Genet..

[41] Xiong K., Zhang J., Zhu Y., Chen L., Ye J. (2019). Zinc doping induced differences in the surface composition, surface morphology and osteogenesis performance of the calcium phosphate cement hydration products. Mater. Sci. Eng. C.

